# Effect of Endoscopic Ureteral Stone Treatment on Kidney Function

**DOI:** 10.7759/cureus.12883

**Published:** 2021-01-24

**Authors:** Volkan Selmi, Sercan Sarı, Mehmet Caniklioğlu, Ünal Öztekin, Mehmet Sakir Taspinar, Levent Işıkay

**Affiliations:** 1 Urology, Yozgat Bozok University, Faculty of Medicine, Yozgat, TUR; 2 Urology, System Hospital, Kayseri, TUR

**Keywords:** ureteroscopy, egfr, kidney function

## Abstract

Introduction: Ureteral stones may have an influence on kidney functions due to postrenal obstruction or urinary infections. Urgent decompression or stone removal is necessary and recommended to prevent further complications in case of severe conditions such as anuria and urosepsis. Although it is believed that ureteral stone removal would result in renal function improvement, there are still unclear points on whether ureteroscopy (URS) can provide benefit as expected and has some adverse effects.

In this study, we aimed to evaluate the alteration of kidney functions of patients who undergo rigid or flexible URS for ureteral stones and find if there are any influencing factors on kidney function alteration.

Materials and Method: We analyzed 126 patients who underwent retrograde intrarenal surgery (RIRS) for renal stones between May 2018 and February 2020 prospectively. The estimated glomerular filtration rate (eGFR) was calculated on the day before the surgery, by modification of diet in renal disease (MDRD) formula. The calculation was repeated and saved three times during follow-up for the same patient; on the day after the operation, on the postoperative 30th day, and the postoperative 90th day. Then, we evaluated the renal function by comparing eGFR and assessed the predicting factors affecting the kidney function.

Results: Preoperative mean eGFR was 82.28 ± 25.20 mL/min/1.73 m^2^ for the study group. Mean eGFR was calculated 90.92 ± 22.97 mL/min/1.73 m^2^ on the first postoperative day, and 94.54 ± 21.95 mL/min/1.73 m^2^ on the third-month follow-up. The mean change in eGFR was 8.63 ± 16.68 mL/min/1.73 m^2^ in the early period and 12.26 ± 21.09 mL/min/1.73 m^2^ in the long-term follow-up period. Fifty-one patients improved on chronic kidney disease (CKD) stage, and 13 deteriorated in three months follow-up.

Conclusion: Removing the stone and relieving the obstruction by ureteroscopic treatment have an alteration on eGFR. Although eGFR improves in the short-term follow-up, amelioration is evident in long-term follow-up, especially in female patients. The other predictive factors for eGFR improvement after URS are the presence of ureteral obstruction and high preoperative serum creatinine levels.

## Introduction

Urolithiasis is one of the significant health problems with increasing prevalence due to the association with other chronic diseases such as diabetes mellitus, obesity, hypertension, metabolic syndrome, hyperlipidemia and epidemiologic factors such as changes in climate, lifestyles and diet [1]. The prevalence of ureter stones has gradually increased, reaching around 20% worldwide, and is the third most common urological problem after urinary tract infection and prostate pathologies [2]. Although stones can remain silent for a long time without any symptoms and pass spontaneously, they also can be symptomatic. Whether ureter stones remain silent for years or can show symptoms such as pain, hematuria and infection, they can damage the kidney and kidney function [3]. Renal colic caused by the obstruction of the ureteral stone is frequent and constitutes a large part of emergency department patient admission [4]. Also, it has some consequences on daily life such as workforce loss, the requirement of immediate treatment because of urosepsis and acute renal failure, and impairment of quality of life [5].

Ureteral stones were mostly treated by open surgery in the past. After the technologic developments and usage of ultrasonic and pneumatic lithotripters, minimally invasive treatment options such as shockwave lithotripsy (SWL) and percutaneous lithotripsy (PNL) took their place. Currently, by enhancement of laser fibers and flexible devices, the trend was towards endoscopic treatment especially flexible ureteroscopic management [6]. Currently, the European Association of Urology (EAU) recommends ureteroscopy (URS) or extracorporeal SWL for stone removal regarding the size and location of the ureteral stone [7]. Ureteral stones may have an influence on kidney functions due to postrenal obstruction or urinary infections. Urgent decompression or stone removal is necessary and recommended to prevent further complications in case of severe conditions such as anuria and urosepsis. Although it is believed that ureteral stone removal would result in renal function improvement, there are still unclear points on whether URS can provide benefit as expected and has some adverse effects.

In this study, we aimed to evaluate the alteration of kidney functions of patients who undergo rigid or flexible URS for ureteral stones and find if there are any influencing factors on kidney function alteration.

## Materials and methods

After the approval from the institutional review board (Decision Number: 2017-KAEK-189_2018.05.30_11), we analyzed 126 patients who underwent retrograde intrarenal surgery (RIRS) for ureteral stones between May 2018 and February 2020 prospectively. Patients who have prior double J catheter (DJ), solitary kidney, a history of chronic renal failure, concomitant kidney stone, ureteral stricture or urinary tract anomaly and missing data during follow-up, were excluded from the study. All patients were evaluated with non-contrast computerized tomography (CT) preoperatively and postoperatively to assess stone-free status. Patients with stones located in the ureter were investigated by complete blood count, urine analysis, urine culture and routine biochemical tests before the operation. The characteristics of the stone and the patient were recorded on the follow-up forms. The sum of the longest dimensions of stones was recorded as the stone size in the case of multiple stones. The stone density was assessed by the CT in Hounsfield Unit (HU). DJ stent was placed in patients in case of mucosal erosion, edema and hematuria. The time between starting endoscopy and end of DJ stent insertion was defined as operation time. Estimated glomerular filtration rate (eGFR) was calculated on the day before the surgery, by modification of diet in renal disease (MDRD) formula:

 (eGFR = 175 x (Serum creatinine)^-1.154^ x (age)^-0.203^ x 0.742 [if female] x 1.212 [if Black]).

The calculation was repeated on the postoperative first day, on the postoperative first month and the third postoperative month. All data were recorded on the follow-up form. The patients were treated with appropriate antibiotics when a urinary tract infection was diagnosed, and all interventions were performed after a sterile urine culture obtained.

Informed consent was obtained from all patients. Intravenous first-generation cephalosporin was administered 30 minutes before the surgery for surgical prophylaxis. All procedures were performed under general anaesthesia. First, the surgeon accessed the ureter by a 7.5 F ureteroscope (Karl Storz®, Tuttlingen, Germany) under the guidance of a guidewire. The 7.5 F ureteroscope was used for diagnostic URS and ureteral dilatation in the RIRS procedure. Ureteral access sheath (Elite Flex®, Ankara, Turkey) was placed in the ureter in all RIRS cases. A 7.5 F flexible ureteroscope (Flex-X2®, Karl Storz, Tuttlingen, Germany) was used for RIRS. A 200 µm laser fiber (Ho YAG Laser; Dornier MedTech®; Munich, Germany/Dornier Med-Tech GmbH, Medilas H20 and HSolvo, Wessling, Germany) was used for laser lithotripsy. The energy of the laser was between 0.8 - 1.5 Joule and 8 - 15 Hz. At the end of the operation, a ureteral stent was placed in all patients. Intraoperative data were recorded, and patients who had no complications were discharged on the postoperative first day.

All patients were checked with a complete blood count, urine analysis and biochemical tests perioperatively. On the first month follow-up, in cases of no residual stone fragments requiring auxiliary interventions, the DJ catheter was displaced after the patient was examined and checked with an X-Ray. All laboratory and screening findings were recorded in the follow-up form. The patients were monitored with CT and routine laboratory tests on the third-month follow-up. The follow-up period for all patients was a minimum of three months after surgery.

All analyses were carried out using the SPSS 25.0 statistical software (IBM Corp. Released 2017. IBM SPSS Statistics for Windows, Version 25.0. Armonk, NY: IBM Corp.). The data distributions were evaluated using the Kolmogorov-Smirnov test. In case of discordance between the graphics and test results, skewness and kurtosis values were considered. Comparisons of preoperative eGFR and postoperative eGFR were performed by paired samples t-test. Other numerical data were analyzed using Student’s t-test, and for categorical data, the chi-square tests were used. The possible influencing factors (age, gender, hydronephrosis, obstruction, stone location, stone volume, stone density, preoperative creatinine level) on renal function alteration, were analyzed with binominal regression analyses; p<0.05 was considered as statistically significant.

## Results

The study was conducted with 126 patients, 36 (28.6%) women, and 90 (71.4%) men. The mean age of the patients was 46.19 ± 1.55 years-old, and the mean body mass index (BMI) was 28.90 ± 4.89. Ten patients had comorbid diseases such as hypertension, diabetes mellitus, coronary artery disease, chronic respiratory disease, and others. Forty-nine patients had proximal ureteral stone, 31 had mid-ureteral stone while 46 had stones located in the distal ureter. Symptoms and signs of ureteral obstruction were seen in 102 patients; however, 94 of these had low grade (grades 1 and 2) hydroureteronephrosis. The mean stone volume was 316.44 ± 277.32 mm^3^. The mean preoperative creatinine level was 1.00 ± 0.33 mg/dL. The demographic data of the patients and stone characteristics are shown in Table 1.

**Table 1:** Characteristics of Patients and Stones and Operation Outcomes BMI: body mass index, eGFR: estimated glomerular filtration rate.

Parameters	n=126
Age (year)	46.19 ± 13.55
Gender (n,%)	
Male	90 (71.4%)
Female	36 (28.6%)
Comorbidity (n,%)	
Hypertension	1 (0.8%)
Diabetes mellitus	1 (0.8%)
Coronary artery disease	7 (5.6%)
Other	1 (0.8%)
Obstruction (+/-) (n,%)	102 (81.0%)/24 (19.0%)
Hydronephrosis (n,%)	
No	24 (19.0%)
Grade 1	41 (32.5%)
Grade 2	53 (42.1%)
Grade 3	7 (5.6%)
Grade 4	1 (0.8%)
Stone localization (n,%)	
Proximal ureter	49 (38.9%)
Mid-ureter	31 (24.6%)
Distal ureter	46 (36.5%)
Number of stones	1.11 ± 0.42
Stone volume (mm^3^)	316.44 ± 277.32
Operation time (minute)	48.56 ± 23.98
Fluoroscopy time (second)	12.47 ± 22.34
Hospitalization (day)	1.67 ± 2.32
Stone-free (%)	93.7%
Complication (n,%)	
Grade I	9 (7.1%)
Grade II	3 (2.4%)
Grade III	1 (0.8%)
Grade IV	0 (0%)
Grade V	0 (0%)
Mean preoperative eGFR (mL/min/1.73 m^2^)	82.28 ± 25.20
Mean postoperative first Day eGFR (mL/min/1.73 m^2^)	90.92 ± 22.97
Mean postoperative third Month eGFR (mL/min/1.73 m^2^)	94.54 ± 21.95

Ninety-three patients (73.8%) underwent semirigid URS while flexible URS was performed in 33 (26.2%). DJ catheter was placed in all patients except two. The mean operative time was 48.56 ± 23.98 minutes. The stone-free rate of the study was 93.7% (n=118). Totally, 13 (10.3%) patients had surgical complications that were mostly low-grade such as fever, hematuria and urinary tract infection. Only one patient required surgical intervention because of steinstrasse. The operation outcomes are also shown in Table 1.

Preoperative mean eGFR was 82.28 ± 25.20 mL/min/1.73 m^2^ for the study group. Mean eGFR was calculated 90.92 ± 22.97 mL/min/1.73 m^2^ on the first postoperative day, and 94.54 ± 21.95 mL/min/1.73 m^2^ on the third-month follow-up. The mean change in eGFR was 8.63 ± 16.68 mL/min/1.73 m^2^ in the early period and 12.26 ± 21.09 mL/min/1.73 m^2^ in the long-term follow-up period. There was a statistically significant improvement in eGFR in the short-term and long-term follow-up when compared to preoperative renal function (p<0.05). Thirty-six patients had improvement, and eight patients had a deterioration of their chronic kidney disease (CKD) stage in the early follow-up period. Fifty-one patients improved on CKD stage, and thirteen deteriorated in three months follow-up. The comparison of eGFR alteration and renal functional outcomes preoperative and postoperative periods are shown in Tables 2 and 3.

**Table 2:** Comparison of eGFR Alteration During Follow-Up eGFR: estimated glomerular filtration rate; Statistical significance in italic.

	Follow-up period		eGFR change	p-Value
Mean eGFR (mL/min/1.73 m^2^)	Preoperative	Postoperative first day		
	82.28 ± 25.20	90.92 ± 22.97	8.63 ± 16.68	<0.05
	Preoperative	Postoperative third month		
	82.28 ± 25.20	94.54 ± 21.95	12.26 ± 21.09	<0.05

**Table 3:** Renal Function Analysis CKD: chronic kidney disease.

	Preoperative (n=126)	Postoperative third month
CKD group (n,%)		
Stage I	50 (39.7%)	76 (60.3%)
Stage II	52 (41.3%)	42 (33.3%)
Stage III	21 (16.7%)	8 (6.3%)
Stage IV	3 (2.4%)	0 (0%)
Stage V	0 (0%)	0 (0%)
CKD stage improvement (n,%)		51 (40.5%)
CKD stage deterioration (n,%)		13 (10.3%)

In the multinominal regression analysis, preoperative creatinine level, gender and presence of ureteral obstruction had significant effects on kidney function improvement. In the analysis, it was observed that; as the preoperative creatinine level increased, the kidney function improvement became more evident, so there was a positive correlation between preoperative creatinine level and recovery (B = 10.43; p = 0.000004). The analysis result was that the improvement in females was more evident than in males (B = 1.84; p = 0.034). In the presence of ureteral obstruction, it is seen that the improvement was more significant than the absence of obstruction. The presence of ureteral obstruction positively correlates with recovery (B = 1.73; p = 0.03).

## Discussion

The size and location of the stone are the most influencing parameters while deciding for the treatment for ureteral stones [8]. Urologists evaluate the patients and perform a surgical intervention according to these factors. Current guidelines recommend URS over SWL for ureteral stones greater than 10 mm and both for ureteral stones smaller than 10 mm [7]. Although semirigid URS can be used for stone removal in the whole ureter, flexible URS is more successful than semirigid URS for stones located in the proximal ureter [9,10]. In this study, we performed flexible URS in 33 of 49 patients who had a proximal ureteral stone. The stones located in the distal and mid-ureter were removed with semirigid URS.

Kirac compared conventional medical treatment, tamsulosin 0.4 mg daily in addition with conventional medical treatment and semirigid URS for distal ureteral stones, and reported that ureteroscopic intervention had higher stone-free rates (95.6%) when compared to others (48.7% and 58.5%, respectively, p<0.0001) [11]. Tugcu et al. achieved a 96.7% stone-free rate for distal ureteral stones treated primarily with semirigid URS and a 95.6% stone-free rate for distal ureteral stones that failed SWL treatment and then underwent semirigid URS [12]. Kartal et al. compared treatment options for proximal ureteral stones and reported stone-free rates as 67.2% on the 15th postoperative day and 94.1% on the third postoperative month after semirigid URS. In the same study, stone-free rates for flexible URS were 89.6% and 97%, respectively [13]. Data in the literature demonstrate that urologists can achieve a high success rate for ureteral stones with semirigid URS. In this study, the stone-free rate was 93.7% which was consistent with previous studies in the literature.

The surgical complication rate of URS has been reported in a wide range. Most of these complications were lower grade and can be managed with conservative treatment [14]. Tepeler et al. evaluated 1208 patients who underwent URS for ureteral stone and reported that the overall complication rate was 12.6% [15]. The most common complications were proximal stone migration (3.9%), mucosal injury (2.8%) and bleeding (1.9%). Urinary tract infection (8%) and stone migration (8%) were the most common complications. In our study, the surgical complication rate (10.3%) was similar to the previous studies.

There are limited studies that reported renal function alteration of ureteral stones and ureteroscopic lithotripsy while several studies evaluated the impact of urolithiasis and its treatment on kidney function [16-19]. Ureteral stones can cause changes in GFR depending on the level of ureteral obstruction they cause. Complete ureteral obstruction decreases eGFR, while partial obstruction has variable effects depending on the severity and duration of the obstruction [20]. In the literature, it is stated that relief of the post-renal obstruction improved renal function. In an animal model, GFR was decreased to <10% of baseline after unilateral ureteral obstruction and returned to the baseline level by 14 days after obstruction relief [21]. In another similarly designed study, GFR was decreased in three hours after the relief of ureteral obstruction but returned to normal level in two weeks period [22]. In this study, both short- and long-term kidney function was improved statistically significantly after ureteroscopic lithotripsy. eGFR change was more in the late period than the early period. Outcomes of this study were consistent with the previous literature findings on ureteral obstruction treatment and kidney function.

Reeves et al. evaluated the effect of ureteroscopic lithotripsy on kidney functions in patients with CKD and reported that the majority of the patients remained stable or improved in their CKD status [19]. Hoarau et al. retrospectively analyzed patients who underwent retrograde intrarenal surgery and stated that 4.9% of patients had significant renal function deterioration while 14.1% had significant renal function improvement [23]. In this study, 40.4% of patients improved CKD status while 10.3% deteriorated, and 49.3% remained stable. These findings were consistent with the data in the literature.

In multinominal logistic regression analysis, gender, preoperative creatinine level and presence of obstruction were significant predictive factors for renal function improvement after ureteroscopic treatment of ureteral stones. Female gender was a predictive factor in our study as same as Piao’s, which explained this was a result of the inhibitor roles of female hormones during inflammatory processes [24]. Higher preoperative serum creatinine level was associated with improvement in renal function after URS. Although Hoarau et al. mentioned multiple procedures and pre-existing CKD were two crucial factors for renal deterioration in univariate analysis, they reported that these factors were not predictive in multivariate analysis. However, they evaluated renal function for the treatment of stones that were located in the kidney, and 63.8% of patients had preoperative ureteral stents. This difference could be explained by the disparity of patient characteristics, and also acute renal insufficiency may be much more predictive than chronic conditions. The other predictive factor was the presence of ureteral obstruction, which may impair the urine flow and cause renal insufficiency. Thus, relief of the obstruction may have a positive impact on renal function [25].

This study has potential limitations. The research was conducted in a single center with a small number of patients. Also, eGFR was the main factor while assessing renal function. Therefore, multicenter prospective randomized controlled trials designed with more specific laboratory, molecular and imaging tests will have a contribution to the literature.

## Conclusions

Endoscopic treatment of ureteral stones is the most effective treatment with a low complication rate. Ureteral obstruction, which was the result of an impacted ureteral stone, deteriorates kidney function. Removing the stone and relieving the obstruction by ureteroscopic treatment have an alteration on eGFR. Although eGFR improves in the short-term follow-up, amelioration is evident in long-term follow-up, especially in female patients. The presence of ureteral obstruction and high preoperative serum creatinine levels are the other predictive factors for improvement after URS. However, further prospective randomized controlled studies conducted with a large number of patients and more specific tests such as cystatin C and radionucleotide scans may confirm the outcomes of this study.
